# Rock1 & 2 Perform Overlapping and Unique Roles in Angiogenesis and Angiosarcoma Tumor Progression

**DOI:** 10.2174/1566524011307010205

**Published:** 2013-01

**Authors:** J Montalvo, C Spencer, A Hackathorn, K Masterjohn, A Perkins, C Doty, A Arumugam, PP Ongusaha, R Lakshmanaswamy, JK Liao, DC Mitchell, BA Bryan

**Affiliations:** 1 Ghosh Science and Technology Center, Department of Biology, Worcester State University, Worcester, Massachusetts, USA; 2 Schepens Eye Research Institute, Harvard Medical School, Boston, Massachusetts, USA; 3 Institute of Biosciences and Technology, Texas A&M Health Science Center, Houston, Texas, USA; 4 Brudnick Neuropsychiatric Research Institute, Neuroscience Program, University of Massachusetts Medical School, Worcester, Massachusetts, USA; 5 Center of Excellence in Cancer Research, Department of Biomedical Sciences, Paul L. Foster School of Medicine, Texas Tech University Health Sciences Center, El Paso, Texas, USA; 6 Vascular Medicine Research Unit, Brigham and Women’s Hospital, Harvard Medical School, Boston, Massachusetts, USA; 7 Genomics Center, Paul L. Foster School of Medicine, Texas Tech University Health Sciences Center, El Paso, Texas, USA

**Keywords:** Angiogenesis, cytoskeleton, endothelial cell, rho kinase, ROCK, vascular endothelial growth factor.

## Abstract

The serine/threonine protein kinase paralogs ROCK1 & 2 have been implicated as essential modulators of angiogenesis; however their paralog-specific roles in endothelial function are unknown. shRNA knockdown of ROCK1 or 2 in endothelial cells resulted in a significant disruption of *in vitro* capillary network formation, cell polarization, and cell migration compared to cells harboring non-targeting control shRNA plasmids. Knockdowns led to alterations in cytoskeletal dynamics due to ROCK1 & 2-mediated reductions in actin isoform expression, and ROCK2-specific reduction in myosin phosphatase and cofilin phosphorylation. Knockdowns enhanced cell survival and led to ROCK1 & 2-mediated reduction in caspase 6 and 9 cleavage, and a ROCK2-specific reduction in caspase 3 cleavage. Microarray analysis of ROCK knockdown lines revealed overlapping and unique control of global transcription by the paralogs, and a reduction in the transcriptional regulation of just under 50% of VEGF responsive genes. Finally, paralog knockdown in xenograft angiosarcoma tumors resulted in a significant reduction in tumor formation. Our data reveals that ROCK1 & 2 exhibit overlapping and unique roles in normal and dysfunctional endothelial cells, that alterations in cytoskeletal dynamics are capable of overriding mitogen activated transcription, and that therapeutic targeting of ROCK signaling may have profound impacts for targeting angiogenesis.

## INTRODUCTION

The RhoA small GTPase and its serine/threonine kinase downstream effector Rho-kinase proteins (ROCK1 & 2) control a wide variety of ubiquitous biological processes including cytoskeleton dynamics, cell movement, proliferation, survival, differentiation, and gene expression [1]. The most understood cellular roles of ROCK proteins are stabilization of actin filaments *via* an inhibitory phosphorylation of LIM kinase, and promotion of cellular contraction and cell substratum contacts *via* increasing myosin motor protein activity through an activating phosphorylation of myosin light chain and an inhibitory phosphorylation of MLC phosphatase [2]. Due to the extensive use of non-selective pharmacological inhibitors of ROCK1 & 2 it is historically believed that these proteins perform overlapping biological roles, however several recent experiments suggest they display distinct functions in development and cell physiology. ROCK1(-/-) mice result in lethality soon after birth, displaying failure of eyelid and ventral body wall closure [3], while ROCK2(-/-) mice experience embryonic lethality due to interuterine growth retardation and placental dysfunction [4]. Viable fertile litters have been reported for ROCK1(+/-) and ROCK2(+/-) mice, however ROCK1(+/-) mice exhibit increased resistance to perivascular fibrosis and reduced vascular injury-induced neointima formation [5, 6], while ROCK2(+/-) mice display decreased platelet endothelial cell adhesion molecule staining of endothelial cells in the lung [7]. Only a handful of recent reports have utilized RNAi technology to singularly disrupt each ROCK paralog *in vitro*, demonstrating unique roles for each protein in the control of actin-cytoskeleton dynamics and cell morphogenesis, migration, cell fate decisions, and extracellular matrix assembly [7-13]. These data collectively suggest that ROCK1 and 2 paralogs perform, at least to some degree, unique biological roles in cell function.

Using *in vivo *and* in vitro* angiogenic assays, several labs have reported that disruption of RhoA/ROCK signaling inhibits vascular endothelial growth factor (VEGF)-mediated endothelial cell activation [7, 14-19]. Moreover, tumor derived endothelial cells display an enhanced ability to organize into capillary networks, correlating with a constitutively high level of RhoA/ROCK signaling [20]. Disruption of ROCK activity in tumor derived endothelial cells normalized network formation to that observed in non-tumor endothelial cells. Our lab has published preliminary data using transiently expressed small interfering RNA (siRNA) technology suggesting that ROCK1 & 2 are both essential for *in vitro* capillary network formation; however the individual contributions of these paralogs to physiological or aberrant endothelial processes are largely unknown. In the present study, we investigate the unique cellular roles of ROCK1 & 2 proteins in endothelial cells and angiosarcoma tumor progression using stably expressed short hairpin RNA (shRNA) plasmids specific for ROCK1 or ROCK2.

## MATERIALS AND METHODS

### Cell Culture and Treatments

MS1 mouse pancreatic endothelial cells (ATCC# CRL-2279), SVR mouse engineered angiosarcoma cells (ATCC# CRL-2280), and B16F1 mouse melanoma cells (ATCC# CRL-6323) were maintained in Dulbecco’s modified Eagle’s media (DMEM) supplemented with 10% fetal bovine serum (FBS), 80 U/ml penicillin, and 50 µg/ml streptomycin C. Cells were treated as indicated with the following concentrations: human recombinant VEGF_165_ (VEGF) (2.5 ng/ml) or Y27632 (10 mM) as previously described [7]. shRNA vectors (SABiosciences) (Table **1**) were transfected using Lipofectamine 2000, and cell pools were stably selected with hygromycin (*MS1 cells*) or puromycin (*SVR cells*).

### Matrigel Angiogenesis Assays

Matrigel capillary network formation assays were performed as previously described [7].

### Tumor Assays

Angiosarcoma tumors were grown using the gelatin sponge-chorioallantoic membrane (CAM) assay according to previously published methods [21]. A hand-cut 1 mm^3^ gelatin sponge (Harvard Apparatus) containing 20,000 dissociated tumor cells was placed onto the CAM of 8 day old fertilized chicken eggs (Charles River Laboratories) and the window was sealed with sterile parafilm. After 8 days growth, tumors were collected, weighed, and photographed on a lightbox. In total, greater than 20 CAMs were collected per condition.

### Immunofluorescence

MS1 cells were grown on glass cover slips, fixed for 10 minutes in 4% paraformaldehyde, and permeabilized for 5 minutes with 0.02% Triton X-100. For actin visualization, coverslips were incubated with rhodamine-conjugated phalloidin for 20 minutes. For ROCK1 & 2 detection, coverslips were stained with antibodies specific to ROCK1 (Abcam #AB58305; Santa Cruz Biotechnology #SC17794) or ROCK2 (Abcam #AB71598; Santa Cruz #SC5561) for 1 hour, and detected with fluorescently labeled secondary antibodies (Invitrogen). As a counterstain, nuclei were detected *via* 5 minute incubation with 4',6-diamidino-2-phenylindole (DAPI) (Sigma). Fluorescence images were captured on a Nikon Eclipse Ti laser scanning confocal microscope.

### RT-PCR

RNA was extracted using TRI Reagent (Molecular Research Center) according to the manufacturer’s instructions. RNA was converted to cDNA using Verso cDNA kit (Thermo Scientific) according to the manufacturer’s instructions. PCR amplification of specific cDNAs was performed using primers designed by Primer Blast (http://www.ncbi.nlm.nih.gov/tools/pri mer-blast). Glyceraldehyde 3-phosphate dehydrogenase (Gapdh) levels were used as a control. Ethidium bromide stained bands were imaged with a GE Image Quant Las4000 imaging station.

### Scratch/Migration Assay

MS1 cells were seeded onto 6-well plates, grown to 100% confluence, and wounded with a sterile pipette tip to remove cells in two perpendicular linear scratches. The progress of migration was photographed immediately following injury and at 12 hr after wounding with a SPOT camera attached to a Nikon Eclipse T150 inverted microscope using SPOT software.

### Underagar Migration Assay

Co-culture migration assays were performed as previously described [22]. Briefly, a (6) well dish was filled with an agarose/culture media mixture containing two wells physically spaced 2.4 mm apart—one containing B16F1 mouse melanoma cells and the other engineered endothelial cells. Co-cultures were incubated for the indicated time course and chemotaxis toward each cell type was quantified using a Nikon Eclipse T150 inverted microscope using SPOT software.

### Proliferation and Survival Assays

For proliferation assays, cells were plated at low confluency and cultured in DMEM (10% FBS). For proliferation assays involving conditioned media, cells were plated at low confluency and cultured in a ratio of 1:1 DMEM (1% FBS):B16F1 conditioned media. For survival assays using cytotoxic agents, cells were plated at 100% confluence in DMEM (10% FBS) and treated with 1 µM cisplatin for 2 days, 1 µM paclitaxel for 5 days, 1 µM busulfan for 2 days, or 10 seconds of 253 nm shortwave ultraviolet radiation using a CL-1000 UV crosslinker (survival was assayed after 24 hours). For survival measurements following serum starvation, cells were plated at 100% confluency in DMEM (0.1% FBS) and allowed to grow for 7 days without media changes. To quantify cell number, MTT assays (Cayman Chemicals) were performed according to the manufacturer’s directions.

### Flow Cytometric Cell Cycle Analysis

Cells were trypsinized, washed in phosphate buffered saline (PBS), and fixed overnight at 4^o^C in a 7:3 ratio of ethanol:PBS. Cells were washed twice in PBS and resuspended in PBS containing 50 µg/ml propidium iodide and 50 µg/ml RNase A. Cells were incubated at 4^o^C overnight and analyzed using an Accuri C6 flow cytometer.

### Western Blotting

Cell lysates were subjected to Western blotting with an antibodies against ROCK1 (Abcam #AB58305), ROCK2 (Abcam #AB71598), phospho-MBS (Abcam #ab59203), MBS (Abcam #ab59235), phospho-cofilin (Cell Signaling #3313), cofilin (Cell Signaling #3318), phospho-ERM (Cell Signaling #3149), ERM (Cell Signaling #3142), cleaved caspase-3 (Cell Signaling #9664), -6 (Cell Signaling #9761), and -9 (Cell Signaling #9509), and tubulin (Santa Cruz #23948), followed by secondary incubation with horse radish peroxidase (HRP) conjugated mono- or poly-clonal antibodies (Invitrogen). HRP was detected with Supersignal West Dura kit (Thermo Scientific) and imaged with a GE Image Quant Las4000 imaging station.

### Microarray Analysis

Six independent biological replicates from each indicated condition were pooled and subjected to triplicate microarray analysis per sample. DNA microarray analysis was performed using the Mouse v2 Whole Genome OneArray (Phalanx Biotech) as previously described [23]. Statistical analysis of the datasets was performed with GeneSpring software using an unpaired t-test (p<0.01). The false discovery rate (FDR) was calculated using the Benjamini Hochberg FDR multiple testing correction. The data discussed in this publication have been deposited in the NIH Gene Expression Omnibus (GEO) and are accessible through GEO accession # GSE34769.

## RESULTS

**Fig. (1):**
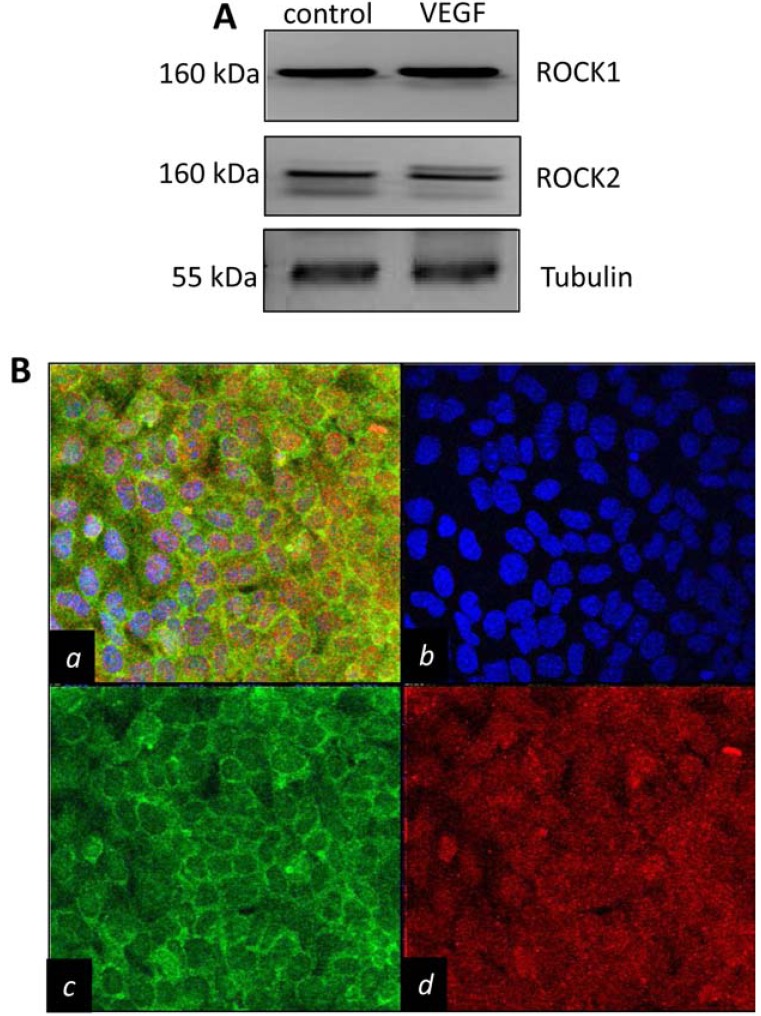
**ROCK1 & 2 are expressed in endothelial cells and exhibit distinct subcellular localization patterns**. (**A**) Western blot analysis of ROCK1 & 2 expression in MS1 endothelial cells grown under standard culture conditions (*control*) or stimulated with 2.5 ng/ml VEGF. (**B**) Fluorescent confocal imaging of ROCK1 & 2 subcellular localization in MS1 endothelial cells with scanning confocal microscopy. (*a=merge; b= DAPI nuclear counterstain; c= ROCK1 localization;d=ROCK2 localization*).

*ROCK paralogs are expressed in endothelial cells and display distinct subcellular localization.* ROCK1 & 2 are ubiquitously expressed protein kinases. To demonstrate the expression of these paralogs in MILE SVEN 1 (MS1) pancreatic islet endothelial cells, which is a well established mouse endothelial line previously developed in Judah Folkman’s laboratory [24], we performed Western analysis to reveal that both ROCK1 & 2 are present at detectable levels (Fig. **1A**). While changes in ROCK1 or 2 subcellular localization was not observed following stimulation of serum starved endothelial cells with 2.5 ng/ml VEGF (Supplementary Fig. **1**), we discovered that in endothelial cells grow under standard culture conditions ROCK1 & 2 localized to punctuate regions, with ROCK1 highly localizing to regions of cell-to-cell adhesion (Fig. **1B**). Co-staining for ROCK1 & 2 revealed that these proteins largely did not co-localize, suggesting they may perform unique functions in endothelial cells.

**Fig. (2):**
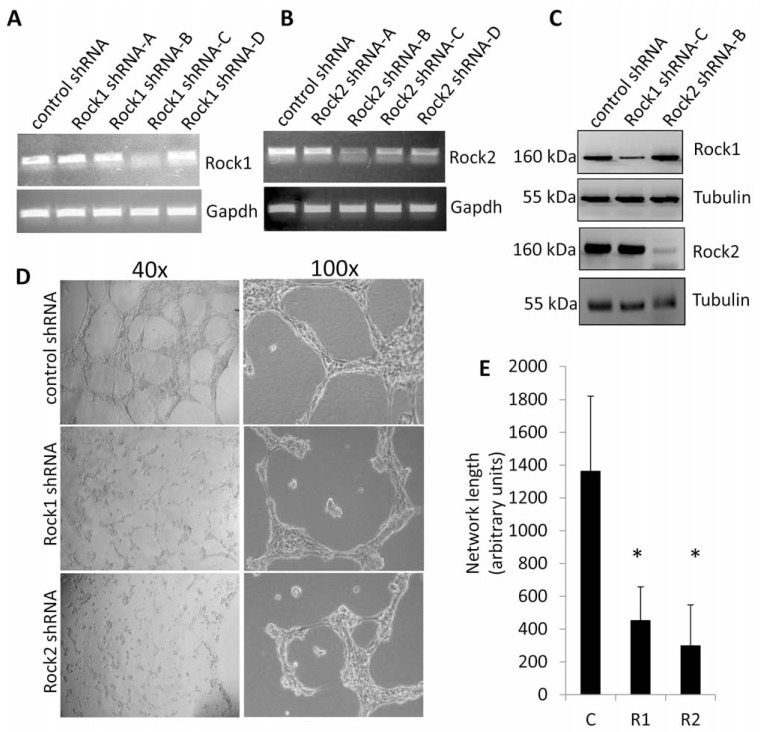
**ROCK1 & 2 are essential for *in vitro* angiogenic network formation**. (**A**, **B**) Semi-quantitative RT-PCR detection of ROCK1/2 steady state mRNA levels in MS1 endothelial cells stably expressing non-targeting control, ROCK1, or ROCK2 shRNA expression vectors. Gapdh transcript levels were used as a control. (**C**) Western blot analysis of ROCK1 & 2 expression in MS1 endothelial cells stably expressing non-targeting control, ROCK1, or ROCK2 shRNA expression vectors. The steady state protein levels of tubulin were used as a loading control. (**D**, **E**) *In vitro* capillary network formation of MS1 endothelial cells stably expressing control, ROCK1, or ROCK2 shRNA expression vectors. Representative images from two independent experiments of the endothelial networks at 40X and 100X are displayed. Data was analysed using Image J software and reported as arbitrary units. (C=control shRNA, R1=ROCK1 shRNA, R2=ROCK2 shRNA; * indicates p<0.05).

*shRNA knockdown of ROCK1 or ROCK2 results in disruption of angiogenic properties of endothelial cells.* MS1 endothelial cells were stably transfected with either non-targeting (control) shRNA or a panel of ROCK1 or ROCK2 shRNA plasmids. To confirm the effectiveness of shRNA knockdown of the ROCK transcripts, semi-quantitative reverse transcriptase polymerase chain reaction (RT-PCR) detecting ROCK1 or ROCK2 steady state mRNA transcript levels was performed on cDNA generated from each culture. Effective knockdown was observed (Fig. **2A, B**), and constructs ROCK1 shRNA-C and ROCK2 shRNA-B (Table **1**) were further confirmed to effectively knock down ROCK proteins levels *via* Western analysis (Fig. **2C**). Moreover, ROCK1 shRNA did not affect ROCK2 steady state protein levels, and ROCK2 shRNA did not affect ROCK1 steady state protein levels (Fig. **2C**), indicating no cross reactivity between the two constructs. We attempted combined double shRNA knockdowns of ROCK1 & 2, but unfortunately failed to obtain surviving endothelial colonies post selection.

We have previously demonstrated that ablation of ROCK1 & 2 kinase activity with the non-selective ROCK1 & 2 pharmacological inhibitor Y27632 resulted in disrupted angiogenesis [7]. To determine the contribution of the individual ROCK paralogs to capillary network formation, the endothelial cell panel stably overexpressing non-targeting, ROCK1, or ROCK2 shRNA plasmids was subjected to matrigel network formation assays. Network formation was quantified after 8 hours, revealing severe disruptions in network assembly in both ROCK1 & 2 shRNA cells compared to non-targeting shRNA cells (Fig. **2D, E**).

**Fig. (3):**
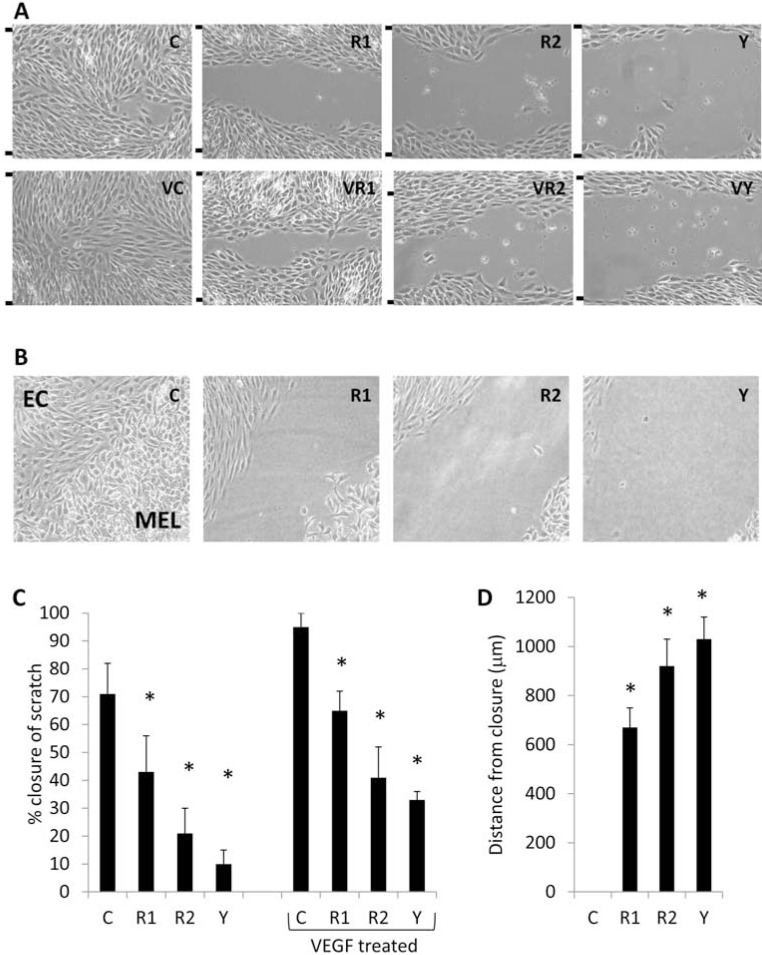
**ROCK1 & 2 are essential for endothelial migration and chemotactic attraction to melanoma tumor cells**. (**A**, **C**) MS1 endothelial cells stably expressing non-targeting control, ROCK1, or ROCK2 shRNA expression vectors, and non-targeting control shRNA MS1 endothelial cells treated with 10 µM Y27632 were grown to 100% confluence. Cells were manually scratched with a P_200_ micropipette tip and subsequently treated with sham or 2 ng/ml recombinant human VEGF. Images of the scratch were collected immediately after (*tick marks on left side of each image represent initial scratch boundary*) and 12 hours after the scratch. Data is reported as the percent closure of the scratch at 12 hours in relation to the initial scratch diameter at time zero. (**B**, **D**) Underagar migration assay using co-cultures of B16F1 mouse melanoma cells (*MEL*) and MS1 endothelial cells (EC) stably expressing non-targeting control, ROCK1, or ROCK2 shRNA expression vectors, and non-targeting control shRNA MS1 endothelial cells treated with 10 µM Y27632. Images of the migrating cells were collected at 3 days after plating. (C=control shRNA, R1=ROCK1 shRNA, R2=ROCK2 shRNA, Y=Y27632; * indicates p<0.05)

*ROCK paralogs display distinct roles in cell organization and migration.* Endothelial cells stably overexpressing non-targeting, ROCK1, or ROCK2 shRNA, or control endothelial cells treated with Y27632 (to disrupt both ROCK1 and ROCK2 kinase activity) were subjected to scratch wound assays in the presence of sham or 2.5 ng/ml VEGF (a strongly pro-migratory endothelial growth factor) and allowed to subsequently migrate/invade into the wounded area over a period of 12 hours. Y27632 treatment of either sham or VEGF treated endothelial cells resulted in a substantial inhibition of wound closure compared to the control or VEGF-treated cells, respectively (Fig. **3A, C**). Cells knocked down for ROCK1 or 2 expression exhibited a retarded migration in both sham and VEGF treated cells, though neither cell line recapitulated the migration defect observed when the kinase activity of both ROCK1 & 2 was inhibited with Y27632.

It is well established that tumor cells secrete numerous pro-angiogenic growth factors including VEGF, fibroblast growth factor (FGF), etc., inducing a strong chemotactic phenotype in endothelial cells. To evaluate the contribution of ROCK paralogs in endothelial chemotaxis and spontaneous migration toward tumor cells, we utilized an underagar co-culture assay whereby the engineered endothelial cells were physically spaced 2.4 mm apart from highly metastatic B16F1 mouse melanoma tumor cells [22]. Three days post-seeding into the wells, the migratory forefronts of non-targeting shRNA endothelial cells and the melanoma cells had collided, while the forefronts of ROCK1 or 2 knockdown cells were 670 +/- 80 and 920 +/- 110 µm apart, respectively (Fig. **3B, D**). Y27632 treated cultures exhibited a failure to migrate far from their initial wells, however, the use of this pharmacological inhibitor does not allow distinction between ROCK-specific effects in endothelial cells versus melanoma cells as both cell types are exposed to its effects under these conditions and we have previously demonstrated that Y27632 induces significant reductions in melanoma migration [25].

While ROCK activity is reportedly essential for migration in a number of cell types, we sought to determine if paralog specificity played a role in the observed phenotypes. It is common that endothelial cells grown to confluence will polarize in culture, recapitulating the alignment these cells take when forming blood vessels. To examine the effect of ROCK paralogs on endothelial polarization, endothelial cells stably overexpressing non-targeting, ROCK1, or ROCK2 shRNA, or control endothelial cells treated with Y27632 were grown to confluency in standard growth conditions. As observed in Fig. (**4A**), cells harboring non-targeting vectors displayed strong cell alignment and polarization, while those knocked down for ROCK1 or 2 expression or inhibited for ROCK kinase activity displayed largely unorganized growth patterns upon confluence.

**Fig. (4):**
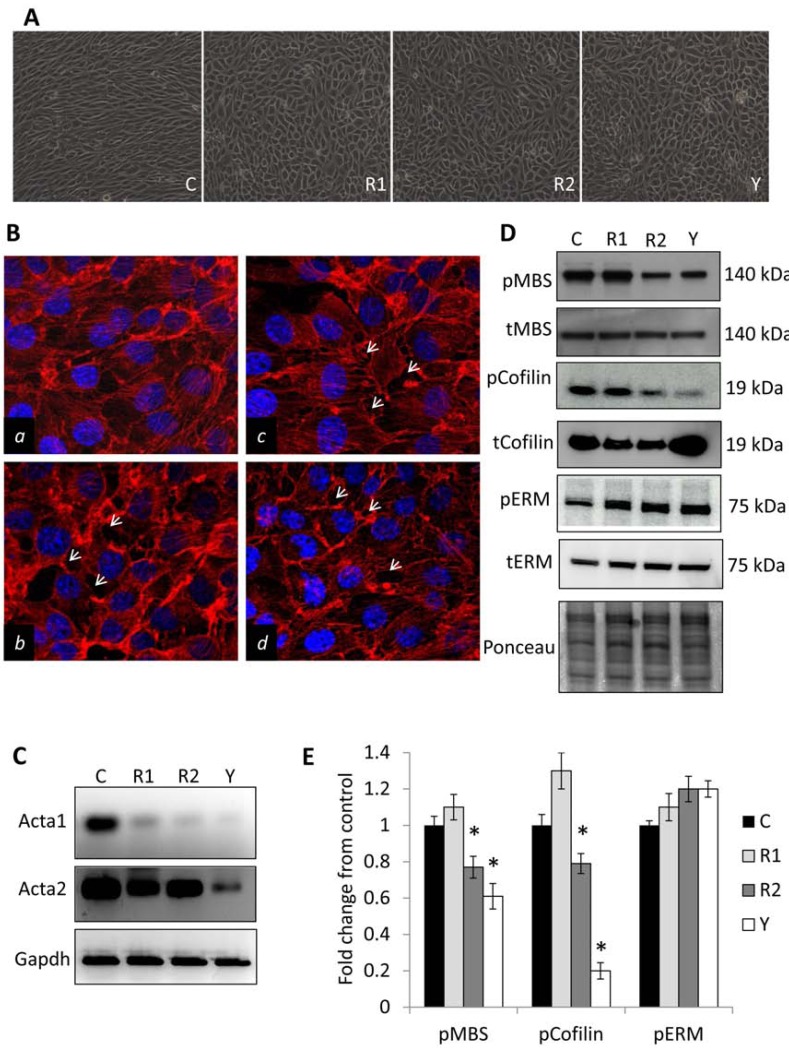
**ROCK1 & 2 display paralog specific cytoskeletal control in endothelial cells**. (**A**) Growth patterns of confluent monolayers of MS1 endothelial cells stably expressing either non-targeting control, ROCK1, or ROCK2 shRNA expression vectors, or non-targeting control shRNA MS1 cells treated with 10 µM Y27632. (**B**) MS1 endothelial cells stably expressing non-targeting control, ROCK1, or ROCK2 shRNA expression vectors, and control shRNA MS1 cells treated with 10 µM Y27632 were grown on glass coverslips for 24 hours. Actin microfilaments in each condition were visualized by rhodamine-conjugated phalloidin staining while nuclei were counterstained with DAPI. Immunofluorescent images were captured with scanning confocal microscopy. (*solid arrows=areas of cell retraction; a=control shRNA, b=Y27632, c= ROCK1 shRNA, d=ROCK2 shRNA*) (**C**) Semi-quantitative RT-PCR analysis of actin alpha 1 (Acta1), actin alpha 2 (Acta2), and Gapdh steady state mRNA expression levels. (**D**) Western blot detection of the phosphorylated (p) and total (t) forms of the myosin binding subunit of myosin phosphatase (MBS), cofilin, and ezrin/radixin/moesin (ERM). Ponceau staining of the membrane was used as a loading control. (**E**) Quantification of the normalized levels of phosphorylated MBS, cofilin, and ERM phosphorylation in each condition. (C=control shRNA, R1=ROCK1 shRNA, R2=ROCK2 shRNA, Y=Y27632; * indicates p<0.05)

To address the contributions of the ROCK paralogs to actin cytoskeletal dynamics in endothelial cells, MS1 cells stably overexpressing non-targeting, ROCK1, or ROCK2 shRNA, or control cells treated with Y27632 were cultured on glass coverslips. Twenty-four hours post-plating, cells were stained with rhodamine-conjugated phalloidin and DAPI, and the actin cytoskeleton was imaged using fluorescent confocal microscopy. Control shRNA cells predominantly displayed well established stress fiber formation throughout the cell body with little to no spacing between individual cells, suggesting strong cell to cell adhesion (Fig. **4B**). In contrast, cells knocked down for ROCK1 or 2 exhibited a significant reduction in stress fiber number and length, multiple regions of strong disorganized actin staining along the cell periphery, and numerous areas where cells appeared to pull away from one another (*solid arrows*). Pharmacological inhibition of ROCK1 & 2 kinase activity with Y27632 displayed limited stress fiber formation accompanied by granularly stained regions reflecting severe actin polymerization issues, as well as strong regions of disorganized actin staining and cell retraction. Interestingly, analysis of cDNA generated from these cell lines revealed that the expression/activity of ROCK1 & 2 is essential for the steady state mRNA expression levels of both alpha 1 & 2 isoforms of actin, which are the major constituent of the cellular contractile apparatus (Fig. **4C**, Supplemental Tables **1** and **2**).

A number of proteins are known targets of ROCK phosphorylation, however the most studied include regulation of actin-myosin contraction *via* an inhibitory phosphorylation of the myosin binding subunit (MBS) of myosin phosphatase [26] and regulation of actin polymer stability *via* inhibition of LIMK2/cofilin-mediated actin severing [27]. Protein lysates were collected from endothelial cells stably overexpressing non-targeting, ROCK1, or ROCK2 shRNA plasmids, or control shRNA cells treated with Y27632 and subjected to Western analysis for the phosphorylated and total (both phosphorylated and non-phosphorylated) forms of MBS, cofilin, and ezrin/radixin/moesin (ERM, a downstream target of ROCK’s kinase activity which has been shown in some cell types to link the cytoskeleton to membrane-bound proteins). As evidenced in Fig. (**4D, E**), ROCK2, but not ROCK1, leads to the reduction in MBS and cofilin phosphorylation, suggesting distinct roles of these paralogs in regulating the endothelial cellular cytoskeleton. No significant change was observed in the normalized levels of ERM phosphorylation in any condition.

**Fig. (5):**
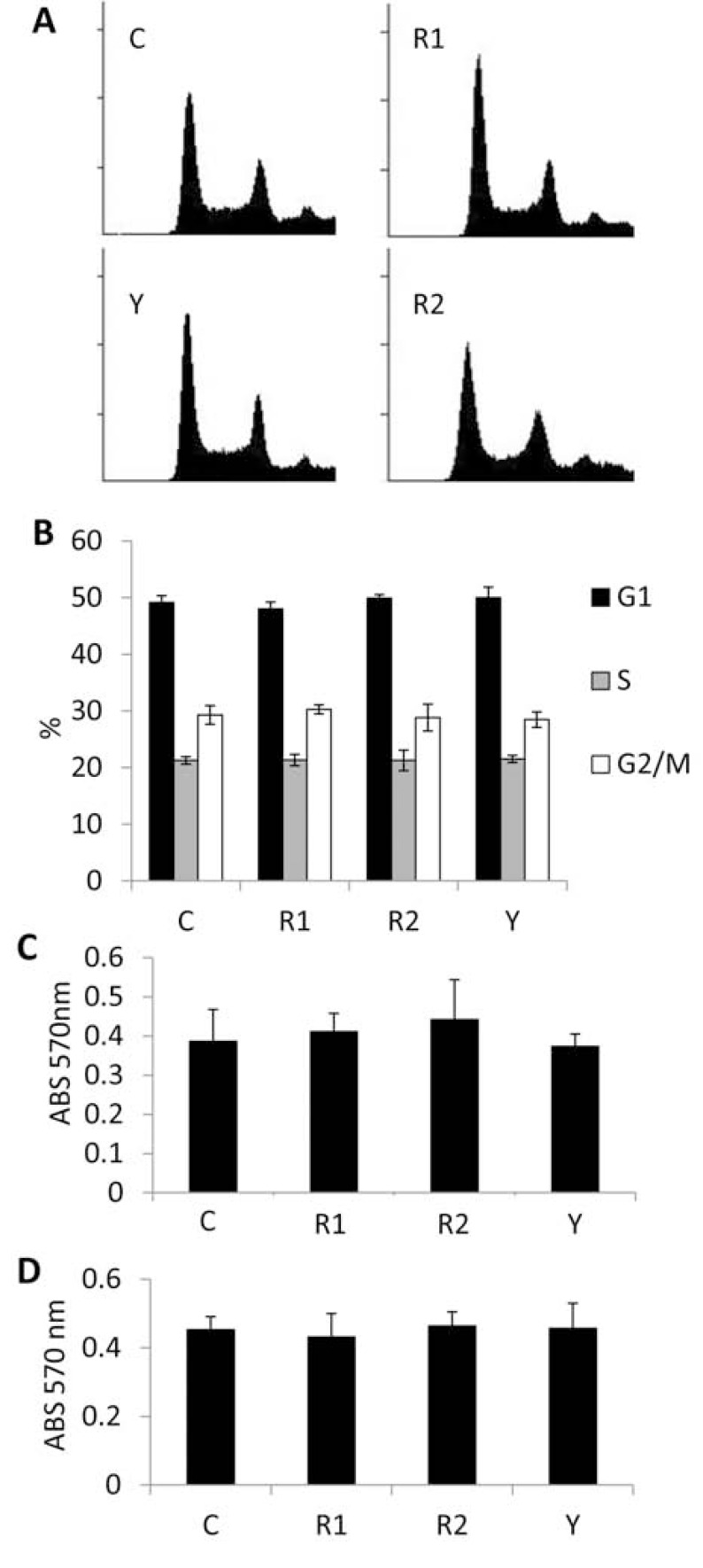
**ROCK paralog control of endothelial proliferation**. (**A**, **B**) MS1 endothelial cells stably expressing either non-targeting control, ROCK1, or ROCK2 shRNA expression vectors, or non-targeting control shRNA MS1 cells treated with 10 µM Y27632 were cultured in standard growth conditions. Flow cytometric analysis of propidium iodide stained MS1 endothelial cells was used to detect progression through each phase of the cell cycle. (**C**) Proliferation of each cell line was accessed 4 days post-plating using the MTT assay. (**D**) MS1 endothelial cells from each condition were cultured in the presence of a 1:1 ratio (standard media:conditioned media collected from B16F1 mouse melanoma cells) and proliferation was accessed 4 days postplating using the MTT assay. (*C=control shRNA, R1=ROCK1 shRNA, R2=ROCK2 shRNA, Y=Y27632*).

**Fig. (6):**
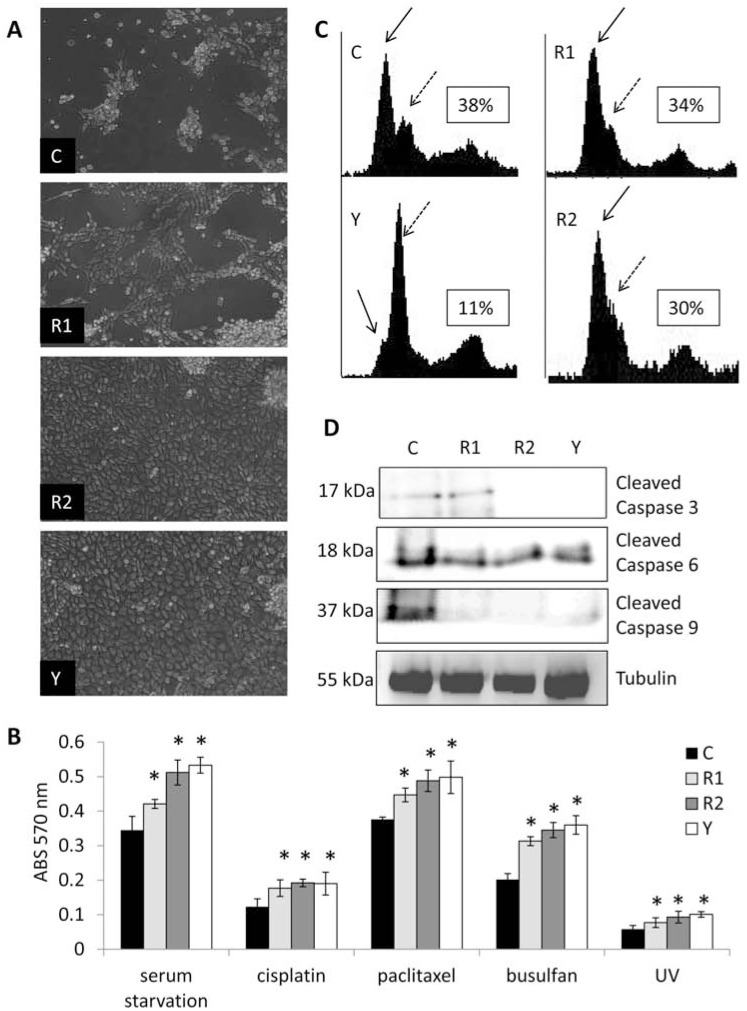
**ROCK1 & 2 display paralog specific control of endothelial cell survival**. (**A**) MS1 endothelial cells stably expressing either non-targeting control, ROCK1, or ROCK2 shRNA expression vectors, or non-targeting control shRNA MS1 cells treated with 10 µM Y27632 were serum starved and photos were taken random fields of view after 7 days. (**B**) Cells were treated with serum starvation, cisplatin, paclitaxel, busulfan, or ultraviolet radiation (UV) as indicated in the Materials and Methods section and cell survival was determined using the MTT assay. (**C**) Flow cytometric analysis of propidium iodide stained serum starved MS1 endothelial cells was used to detect the amount of the sub-G1 apoptotic peak. (*inset=percent apoptotic cells; solid arrow=sub-G1 apoptotic peak, dashed arrow=G1 peak*) (**D**) Western blot analysis of cleaved caspase 3, 6, & 9. The steady state protein level of tubulin was used as a loading control. (C=control shRNA, R1=ROCK1 shRNA, R2=ROCK2 shRNA, Y=Y27632; * indicates p<0.05).

*ROCK paralog control of endothelial proliferation and survival.* It has been reported that ROCK signaling promotes cell cycle progression into the S phase through a diverse array of downstream targets [28], however our data indicate that in endothelial cells grown under standard culture conditions, loss of ROCK expression or activity exerts no effect on progression through the cell cycle (Fig. **5A, B**) or overall proliferation rate (Fig. **5C**). Moreover, addition of B16F1 melanoma cell conditioned media failed to affect proliferation alterations in the knockdown lines compared to the control (Fig. **5D**). However, subjecting endothelial cells stably overexpressing non-targeting, ROCK1, or ROCK2 shRNA, or control cells treated with Y27632 to serum starvation or cytotoxic treatment led to marked increases in cell survival in cells knocked down for ROCK expression or with inhibition of ROCK kinase activity (Fig. **6A, B**). Moreover, flow cytometric analysis of propidium iodide stained cells under serum starvation conditions revealed significant reductions in the sub-G1 apoptotic peak when ROCK expression or activity was reduced (Fig. **6C**). Under all conditions tested, ROCK2 knockdown cells displayed consistently higher survival rates and less apoptosis compared to ROCK1 knockdown cells. To explain this, we performed Western analysis for caspase cleavage products on lysates collected from each serum starved cell line. As demonstrated in Fig. (**6D**), knockdown of ROCK1 & 2 as well as inhibition of ROCK kinase activity led to a significant reduction in the cleavage products of effector caspases 6 and 9 levels. Interestingly, no difference was observed in the levels of cleavage products for caspase 3 in the endothelial cells harboring non-targeting *vs* ROCK1 shRNA vectors, while ROCK2 knockdown and Y27632 treatment exhibited a marked reduction, suggesting distinct roles for the ROCK paralogs in the regulation of apoptosis.

**Fig. (7):**
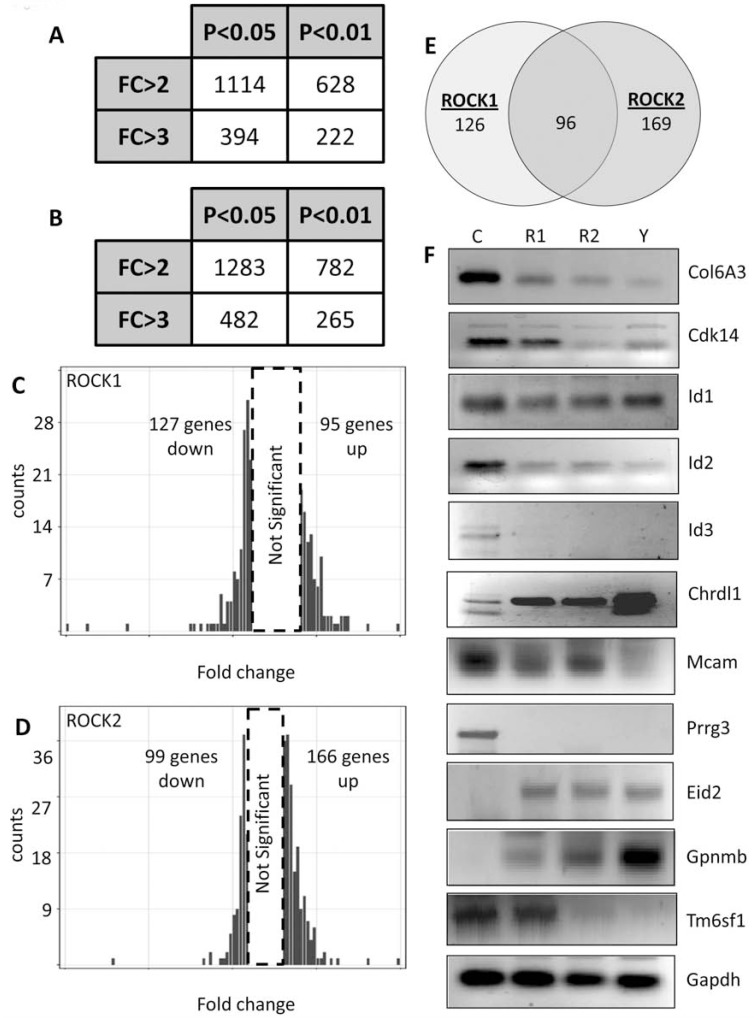
**ROCK1 & 2 control the expression of overlapping and distinct subsets of target transcripts**. (**A**, **B**) MS1 endothelial cells stably expressing either non-targeting control, ROCK1, or ROCK2 shRNA expression vectors were subjected to microarray analysis of over 24,000 transcripts. Illustration of the number of genes that were statistically altered in cells harboring ROCK1 shRNA (**A**) and ROCK2 shRNA (**B**) compared to the control at differing statistical stringencies. (*FC=fold change*) (**C**, **D**) Histograms representing the distribution of transcripts that were statistically altered compared to the control at the highest stringency utilized (FC>3, p<0.01) in ROCK1 (**C**) or ROCK2 (**D**) shRNA harboring endothelial cells. (**E**) Venn diagram illustrating the number of transcripts whose expression is shared by or unique to ROCK1 & 2 (FC>3, p<0.01). (**F**) Semi-quantitative RTPCR confirmation of selected transcripts identified in the microarray analysis. (*C=control shRNA, R1=ROCK1 shRNA, R2=ROCK2 shRNA, Y=Y27632*)

*ROCK paralog control of global endothelial cell transcription.* Despite the primary focus of most published studies emphasizing ROCK’s regulation of the phosphoproteome, a handful of reports using non-selective pharmacological inhibitors of ROCK1 & 2 activity have demonstrated large scale alterations in global gene expression of *in vivo* melanoma tumors and *in vitro* epithelial, endothelial, and mesenchymal cells [23, 29-31], though no distinction has been made regarding the individual ROCK paralog contributions to gene expression. To determine the effects of ROCK paralog activity on the endothelial global transcriptome, we cultured endothelial cells harboring non-targeting control, ROCK1, or ROCK2 shRNA vectors at 100% confluence and performed microarray analysis on over 24,000 genes. Genes were considered statistically significant if their expression changed by greater than three-fold (p<0.01). Using these cutoffs, we identified 222 and 265 genes whose expression was significantly altered in ROCK1 & 2 shRNA cells, respectively, compared to non-targeting shRNA control cells (Fig. **7A-D**, Supplemental Tables **1** and **2**, GEO accession # GSE34769). 126 gene expression changes were unique to ROCK1 knockdown, while 169 gene expression changes were unique to ROCK2 knockdown, indicating both overlapping and non-overlapping roles of ROCK paralogs in the regulation of gene transcription (Fig. **7E**). A subset of the identified gene expression changes were confirmed at the mRNA level with RT-PCR (Fig. **7F**).

**Fig. (8):**
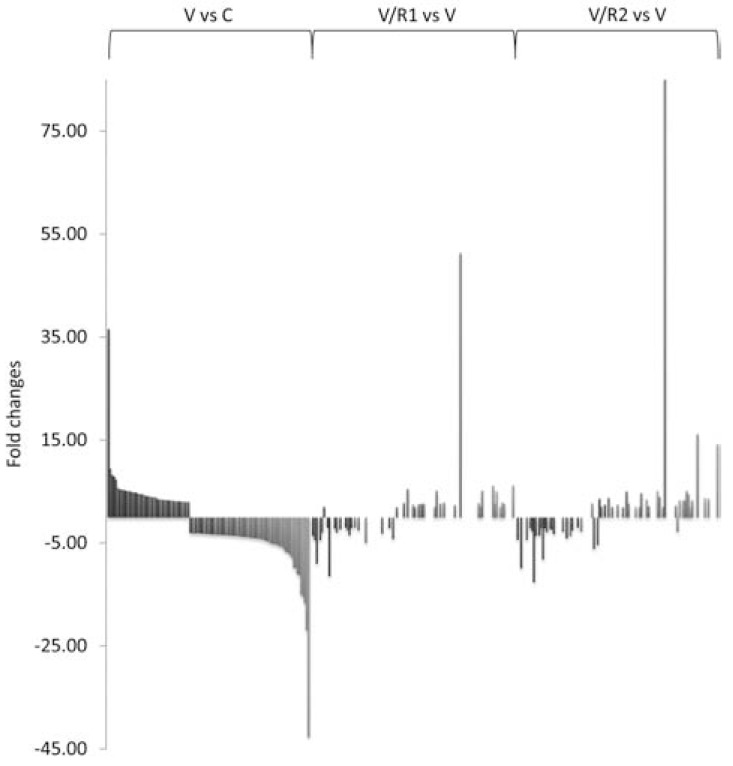
**ROCK1 & 2 modulate the expression just under half of VEGF-responsive genes**. MS1 endothelial cells stably expressing either non-targeting control, ROCK1, or ROCK2 shRNA expression vectors were treated with sham or 2.5 ng/ml VEGF and subjected to microarray analysis of over 24,000 transcripts. 114 genes were identified whose expression was altered by more than three-fold (p≤0.01) in VEGF-treated endothelial cells harboring the non-targeting control shRNA vector compared to sham treatment (*V vs C*). ROCK1 (*V/R1 vs V*) or 2 (V/R2 *vs* V) shRNA harboring endothelial cells treated with VEGF exhibited an ablation of transcript expression in 40% and 49%, respectively, of the identified VEGF responsive genes.

As VEGF strongly regulates large-scale gene expression in endothelial cells and previous studies have indicated that ROCK signaling is essential for VEGF-mediated angiogenesis, we sought to elucidate the individual contributions of ROCK1 & 2 to the control of VEGF-driven global gene transcription in endothelial cells. To address this, endothelial cells stably expressing non-targeting control, ROCK1, or ROCK2 shRNA were treated with sham or 2.5 ng/ml VEGF for 12 hours, and global gene expression changes were analyzed using microarrays. We identified 114 genes whose expression was altered by more than three-fold (p≤0.01) in VEGF-treated endothelial cells harboring the non-targeting control shRNA vector compared to sham treatment (Supplemental Table **3**). A 2-fold or greater ablation of gene expression occurred in approximately 40% (for VEGF-treated ROCK1 shRNA endothelial cells) and 49% (for VEGF-treated ROCK2 shRNA endothelial cells) of the identified VEGF responsive genes (Fig. **8**, Supplemental Table **3**, GEO accession # GSE34769).

**Fig. (9):**
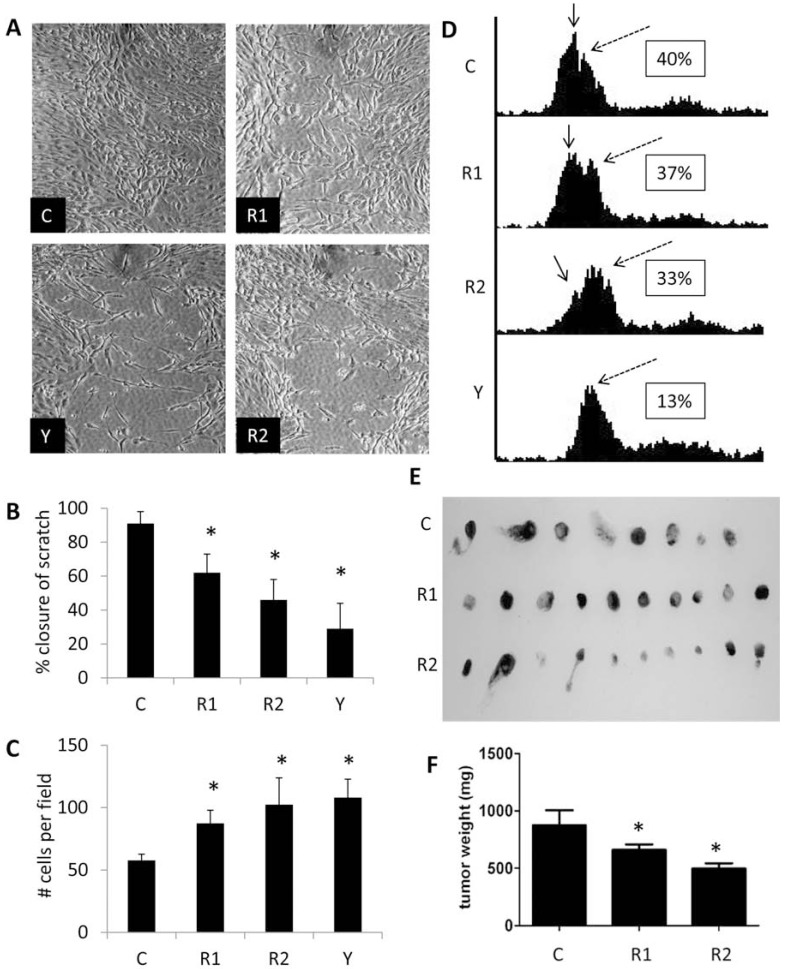
**ROCK paralogs are essential for multiple aspects of angiosarcoma cellular function and tumor formation**. (**A**, **B**) SVR angiosarcoma cells stably expressing non-targeting control, ROCK1, or ROCK2 shRNA expression vectors, and non-targeting control shRNA SVR cells treated with 10 µM Y27632 were grown to 100% confluence. Cells were manually scratched with a P_200_ micropipette tip. Images of the scratch were collected immediately after (*not shown*) and 12 hours after the scratch. Data is reported as the percent closure of the scratch at 12 hours in relation to the initial scratch diameter at time zero. (**C**) The SVR angiosarcoma cell lines were serum starved (0.1% FBS) for 7 days and cell survival was accessed using the MTT assay. (**D**) Flow cytometric analysis of propidium iodide stained serum starved SVR angiosarcoma cells was used to detect the amount of the sub-G1 apoptotic peak. (*inset=percent apoptotic cells; solid arrow=sub-G1 apoptotic peak, dashed arrow=G1 peak*) (**E**, **F**) A gelatin sponge containing 20,000 SVR angiosarcoma cells stably expressing non-targeting control, ROCK1, or ROCK2 shRNA expression vectors was placed on the CAM membrane of 8 day old fertilized chicken eggs. Tumors were collected after 8 days of tumor growth, photographed, and weighed. (C=control shRNA, R1=ROCK1 shRNA, R2=ROCK2 shRNA, Y=Y27632; * indicates p<0.05).

*ROCK paralogs are essential for multiple aspects of angiosarcoma cellular function and tumor formation.* Given the current interest in targeting Rho/ROCK signaling in tumor progression, metastasis, and angiogenesis, we sought to expand our findings from endothelial cells to that of tumors of vascular origin. Similar to our findings in endothelial cells, knockdown of the ROCK paralogs or pharmacological inhibition of ROCK activity with Y27632 led to reductions in SVR mouse angiosarcoma cell migration and enhanced survival following serum starvation (Fig. **9A-D**). Moreover, no changes in proliferation rates or cell cycle progression were observed in the knockdowns or pharmacological treatment (*data not shown*). Using a tumor xenograft system, SVR cells stably expressing non-targeting control, ROCK1, or ROCK2 shRNA plasmids were seeded onto 1 mm^2 ^gelatin sponges and implanted onto the chorioallantoic membrane of 8 day post-fertilization chicken eggs. Tumors were allowed to grow for an additional 8 days and subsequently collected, weighed, and photographed. Tumor weight was significantly reduced in ROCK1 & 2 knockdown tumors compared to control tumors, with ROCK2 shRNA tumors displaying smaller tumors than those formed by ROCK1 knockdown cells (Fig. **9E, F**).

## DISCUSSION

ROCK signaling is rapidly activated upon VEGF stimulation of endothelial cells and controls a diverse number of endothelial processes [7], however previous studies utilized non-selective pharmacological inhibitors of ROCK1 & 2 kinase activity, thus promiscuously inhibiting both paralogs. The two ROCK paralogs share 65% identity overall and 92% identity in their kinase domains [32] and ROCK1 & 2 knockout mice display unique phenotypes [3, 5-7], suggesting that their regulation and signaling may be divergent to a measurable degree. Indeed, comparisons of RNAi knockdowns of the individual ROCK paralogs across a handful of cell types uncovered unique roles for each paralog in actin organization, migration, cellular morphogenesis, cell cycle progression, apoptosis, and extracellular matrix assembly [8-10, 12, 13, 33].

In this study, we analyzed the ROCK paralog specific roles in endothelial and angiosarcoma function using shRNA knockdown technology and demonstrate that ROCK1 & 2 are important for endothelial migration, cytoskeletal regulation, survival, network formation, and global gene expression, and in many cases exhibit paralog-specific regulation of these processes. Our first indication that these paralogs may play non-overlapping roles in endothelial physiology stemmed from our immunofluorescence data indicating that ROCK1 & 2 proteins display unique subcellular localization. Further experiments revealed that while loss of either ROCK paralog resulted in no change in endothelial proliferation rates, substantial increases in cell viability were observed when these cells were challenged with cytotoxic insults such as serum starvation, chemotherapy, or UV irradiation. Primarily using pharmacological inhibition of total ROCK activity, numerous studies have previously implicated these paralogs as key regulators of cell proliferation and survival, however their role in these processes are largely pleiotropic across diverse cell lines [28]. For instance, ROCK signaling acts in a pro-apoptotic manner through promoting caspase cleavage, regulating both intrinsic and extrinsic apoptotic regulators, and enhancing the activation of the pro-survival phosphoinositide 3-kinase (PI3K) pathway [28]. In contrast, ROCK activity is essential for cell cycle progression *via* its control of the expression of cyclins, cyclin dependent kinases (CDKs), and numerous other cell cycle regulators [28]. Further studies are necessary to determine the genetic/signaling factors at play which determine whether ROCK paralogs promote or inhibit cell survival. Our data indicate that loss of ROCK1 or 2 significantly reduces the migration rate of both unstimulated and growth factor stimulated endothelial cells through disruption in cytoskeletal dynamics and the activation status of key cytoskeletal regulators. Interestingly, studies using non-specific pharmacological inhibitors of ROCK1 & 2 have stated that ROCK activity is essential for the phosphorylation of the actin regulating MBS and cofilin proteins [27], however our data in endothelial cells indicates that only ROCK2 is essential for MBS and cofilin phosphorylation. Indeed, similar findings are reported for vascular smooth muscle cells where ROCK2, but not ROCK1, binds and phosphorylates MBS [34]. Moreover, the coiled-coil region of ROCK2 (amino acids 338-750) that interacts with MBS shares only 58% homology with the equivalent region in ROCK1 and this domain of ROCK1, but not ROCK2, reportedly binds to RhoE and PDK1 [35, 36] indicating protein-protein interaction specificity between the two paralogs. These findings demonstrate the limitations of generally classifying ROCK1 & 2 activity based solely on data collected from non-selective pharmacological inhibitors. One may ask which ROCK paralog plays the major function in endothelial physiology, however our experimental data suggest that both paralogs are vitally important as loss of either paralog dramatically inhibits multiple cellular processes.

Over the past decade, the almost exclusive majority of studies have examined the role of ROCK protein regulation of the phosphoproteome. While many reports reveal singular ROCK-mediated changes in gene expression, a limited number of studies have examined the dramatic alterations in global gene transcription following pharmacological inhibition of total ROCK activity [23, 29-31]. We expand on these studies by demonstrating that ROCK1 & 2 knockdown leads to large scale changes in the endothelial transcriptome and these paralogs perform non-overlapping protein-specific roles in the regulation of a significant portion of these genes. Mechanotransduct-ion studies demonstrate that mechanical forces exerted from the cellular microenvironment or by alterations in cell shape impinge on the cytoskeleton and other cellular components to produce global changes in cellular function altering cellular decisions for proliferation, differentiation, or death [37, 38]. Indeed, knockdown of ROCK 1 & 2, which are two of the central regulators of cytoskeletal dynamics, effectively ablates just shy of 50% of VEGF-driven gene expression. These data suggest the possibility that mechanotransduction mediated through ROCK signaling cascades is capable of modulating chemically derived growth factor cell stimulation, and may help explain the ambiguity observed by signaling cascades across cell types that is often attributed to micro-environment effects. This observation has profound implications for cancer therapy given that the initiation of angiogenesis in solid tumors begins with hypoxic tumor secretion of VEGF to serve as a chemoattractant for endothelial cell infiltration into the tumor.

Proteins involved in the Rho-signaling cascade are significantly elevated in a variety of cancers [39-43], and pharmacological inhibition of ROCK activity shows preclinical efficacy in the treatment of *in vivo* malignancies including prostate, breast, glioma, melanoma, and human papillomavirus infected tumors [25, 44-47]. Given that ROCK signaling regulates both solid tumor progression and angiogenesis, we sought to examine its role in angiosarcoma solid tumors which are formed extensively by dysfunctional, aberrantly proliferating endothelial cells. We report that knockdown of either ROCK paralog results in a significant reduction in angiosarcoma tumor volume. These findings are the first study to compare side-by-side the contributions of ROCK1 & 2 to solid tumor formation. Indeed, therapeutic targeting of ROCK activity in angiosarcomas and other solid tumors could potently disrupt two major processes essential for tumor progression and metastasis: migration and angiogenesis.

## Figures and Tables

**Table 1.** shRNA Construct Sequences

Gene	Sequence

*ROCK1*	A) GGAGGATGAAGTTAAGAATCT
	B) GGCTGGAAGAAACAGTATGTT
	C) GCGCAATTGGTAGAAGAATGT
	D) CGGGAGTTACAAGATCAACTT

*ROCK2*	A) GCAGCTATTAAAGCACAGTTT
	B) AACCAACTGTGAGGCATGTAT
	C) AGAGCAGTCCAACCCTTACAT
	D) GGAACAGAAGTGCAAATCTAT

Scrambled	GGAATCTCTCATTCGATGCATAC

## ABBREVIATIONS

ROCK: = Rho-associated, coiled-coil kinase
LIM: = Lin11, Isl1, and Mec3
MLC: = Myosin light chain
RNAi: = RNA inhibition
siRNA: = Small interfering RNA
shRNA: = Small hairpin RNA
MS1: = MILE SVEN
VEGF: = Vascular endothelial growth factor
RTPCR: = Reverse transcriptase polymerase chain reaction
cDNA: = Complementary DNA
FGF: = Fibroblast growth factor
MBS: = Myosin binding subunit
ERM: = Exrin/radixin/moesin
CAM: = Chorioallantoic membrane
