# Supplementary material for: Rock1 & 2 Perform Overlapping and Unique Roles in Angiogenesis and Angiosarcoma Tumor Progression
Source: Curr Mol Med. 2013 Jan;13(1):205–19. doi: 10.2174/1566524011307010205 (PMC3580831; doi:10.2174/1566524011307010205)
Supplement: Supplementary file 1 [file CMM-13-205_SD1.pdf]

# SUPPLEMENTARY MATERIAL

## Rock1 & 2 Perform Overlapping and Unique Roles in Angiogenesis and Angiosarcoma Tumor Progression

John Montalvo[1,§], Carrie Spencer[1,2,§], Alexander Hackathorn[1], Katherine Masterjohn[1], Ashley Perkins[1,3], Christopher Doty[1,4], Arunkumar Arumugam[5], Pat P. Ongusaha[6], Rajkumar Lakshmanaswamy[5], James K. Liao[6], Dianne C. Mitchell[7], Brad A. Bryan[1,5]

[1]*Ghosh Science and Technology Center, Department of Biology, Worcester State University, Worcester, Massachusetts, USA*

[2]*Schepens Eye Research Institute, Harvard Medical School, Boston, Massachusetts, USA*

[3] *Institute of Biosciences and Technology, Texas A&M Health Science Center, Houston, Texas, USA*

[4]*Brudnick Neuropsychiatric Research Institute, Neuroscience Program, University of Massachusetts Medical School, Worcester, Massachusetts, USA*

[5]*Center of Excellence in Cancer Research, Department of Biomedical Sciences, Paul L. Foster School of Medicine, Texas Tech University Health Sciences Center, El Paso, Texas, USA*

[6]*Vascular Medicine Research Unit, Brigham and Women's Hospital, Harvard Medical School, Boston, Massachusetts, USA*

[7]*Genomics Center, Paul L. Foster School of Medicine, Texas Tech University Health Sciences Center, El Paso, Texas, USA*

**Supplemental Table 1. Top Genes Regulated by ROCK1 shRNA Transfected MS1 Endothelial Cells Relative to Control shRNA Transfected MS1 Endothelial Cells**

| Gene Symbol | Accession Number | Fold Change | p-Value |
|---|---|---|---|
| Eid2 | NM_198425.2 | 85.14 | 2.53E-04 |
| Gpnmb | NM_053110.4 | 34.80 | 1.33E-05 |
| Ripk3 | NM_001164107.1 | 30.21 | 7.81E-05 |
| Lilrb4 | NM_013532.2 | 29.77 | 1.63E-04 |
| Gmps | NM_001033300.2 | 28.52 | 2.94E-08 |
| Mir33 | NR_029804.1 | 26.78 | 6.65E-04 |
| Cdh2 | NM_007664.4 | 17.15 | 5.92E-06 |
| Inpp5d | NM_001110192.1 | 16.62 | 8.82E-05 |
| Sprr1a | NM_009264.2 | 12.94 | 2.54E-05 |
| Hoxc6 | NM_010465.2 | 12.87 | 4.20E-04 |
| Usp18 | NM_011909.2 | 12.11 | 2.27E-06 |
| Hs6st2 | NM_001077202.1 | 11.59 | 2.74E-06 |
| Pdx1 | NM_008814.3 | 10.83 | 3.93E-04 |
| Kcne3 | NM_020574.5 | 10.52 | 2.71E-05 |
| Gm10632 | XR_106159.1 | 9.32 | 4.42E-04 |
| Enah | NM_001083120.1 | 9.30 | 1.20E-04 |
| Chrdl1 | NM_001114385.1 | 9.03 | 2.00E-05 |
| 1600029D21Rik | NM_029639.2 | 8.09 | 4.25E-08 |
| Paox | NM_153783.4 | 7.99 | 2.96E-05 |

**(Supplemental Table 1) contd…..**

| Gene Symbol | Accession Number | Fold Change | p-Value |
|---|---|---|---|
| Rtp4 | NM_023386.5 | 7.66 | 1.13E-05 |
| Msmp | NM_001099314.1 | 7.61 | 9.34E-07 |
| Nefl | NM_010910.1 | 7.27 | 6.14E-07 |
| Edn1 | NM_010104.3 | -6.67 | 2.39E-05 |
| Ttc18 | NM_001163638.1 | -6.91 | 4.20E-05 |
| Rn7sk | NR_030687.1 | -7.01 | 6.43E-04 |
| Slco1a5 | NM_130861.2 | -7.12 | 5.38E-05 |
| Vwf | NM_011708.3 | -7.19 | 5.48E-05 |
| Epas1 | NM_010137.3 | -7.22 | 1.12E-05 |
| Cryab | NM_009964.2 | -7.28 | 9.87E-07 |
| Klk8 | NM_008940.2 | -7.97 | 1.50E-05 |
| Pspn | NM_008954.2 | -8.14 | 5.87E-05 |
| Icam1 | NM_010493.2 | -8.51 | 3.24E-06 |
| Pip5kl1 | NM_198191.2 | -8.89 | 2.39E-04 |
| Zfp334 | NM_178411.3 | -10.63 | 1.87E-04 |
| Fbln2 | NM_001081437.1 | -10.73 | 6.87E-06 |
| Mcam | NM_023061.2 | -11.24 | 2.11E-07 |
| Msln | NM_018857.1 | -11.41 | 1.37E-05 |
| Fos | NM_010234.2 | -11.61 | 4.25E-06 |
| D0H4S114 | NM_001109988.1 | -11.82 | 8.78E-07 |
| Acta1 | NM_009606.2 | -13.85 | 7.37E-05 |
| Cyp1b1 | NM_009994.1 | -19.18 | 4.53E-07 |
| Lcn2 | NM_008491.1 | -22.06 | 9.53E-07 |
| Id3 | NM_008321.2 | -34.29 | 9.34E-07 |
| Prrg3 | NM_001081135.1 | -36.02 | 3.28E-06 |
| Fhl1 | NM_001077361.1 | -41.95 | 2.11E-06 |
| Yeats2 | NM_001033237.2 | -834.90 | 3.47E-08 |
| Steap2 | NM_001103156.1 | -1896.57 | 1.39E-04 |

**Supplemental Table 2. Top Genes Regulated by ROCK2 shRNA Transfected MS1 Endothelial Cells Relative to Control shRNA Transfected MS1 Endothelial Cells**

| Gene Symbol | Accession Number | Fold Change | p-Value |
|---|---|---|---|
| Csf2 | NM_009969.4 | 690.08 | 2.83E-05 |
| Cxcl2 | NM_009140.2 | 70.43 | 1.47E-10 |
| Eid2 | NM_198425.2 | 69.17 | 3.07E-04 |
| 1810011O10Rik | NM_026931.2 | 40.88 | 6.11E-06 |
| Serpina3m | NM_009253.2 | 38.69 | 1.28E-04 |
| Gpnmb | NM_053110.4 | 30.18 | 1.46E-05 |
| Ripk3 | NM_001164107.1 | 29.59 | 7.76E-05 |
| Hs6st2 | NM_001077202.1 | 20.94 | 1.40E-06 |
| Il20rb | NM_001033543.3 | 20.44 | 4.57E-07 |

**(Supplemental Table 2) contd…..**

| Gene Symbol | Accession Number | Fold Change | p-Value |
|---|---|---|---|
| H2-K2 | NR_004446.1 | 17.97 | 1.61E-05 |
| Gmps | NM_001033300.2 | 17.85 | 8.40E-08 |
| Cdh2 | NM_007664.4 | 17.84 | 5.56E-06 |
| Usp18 | NM_011909.2 | 17.10 | 8.98E-07 |
| Cxcl10 | NM_021274.1 | 16.90 | 2.99E-06 |
| Tnfaip3 | NM_001166402.1 | 15.34 | 5.00E-08 |
| Gap43 | NM_008083.2 | 15.24 | 2.04E-06 |
| Rxfp1 | NM_212452.1 | 14.92 | 4.13E-04 |
| Cd69 | NM_001033122.3 | 14.84 | 4.09E-04 |
| Slc2a3 | NM_011401.4 | 14.34 | 6.87E-05 |
| Inpp5d | NM_001110192.1 | 13.99 | 8.69E-05 |
| Irf7 | NM_016850.2 | 12.93 | 2.38E-07 |
| Oasl1 | NM_145209.3 | 12.62 | 1.09E-06 |
| Lphn1 | NM_181039.2 | -4.91 | 1.93E-04 |
| Vwf | NM_011708.3 | -4.96 | 7.79E-06 |
| Ssr1 | NM_025965.3 | -5.23 | 2.07E-06 |
| Mras | NM_008624.3 | -5.25 | 1.25E-05 |
| Htra4 | NM_001081187.3 | -5.31 | 1.89E-04 |
| Mgat4c | NM_001162368.1 | -5.72 | 3.53E-05 |
| Ttc18 | NM_001163638.1 | -5.99 | 3.85E-06 |
| Cbfa2t3 | NM_001109873.1 | -5.99 | 8.18E-07 |
| Pid1 | NM_001003948.2 | -6.36 | 1.38E-04 |
| Fhl1 | NM_001077361.1 | -6.40 | 1.11E-05 |
| Ly6g6d | NM_033478.2 | -6.48 | 1.98E-04 |
| Ptplb | NM_023587.2 | -6.75 | 3.46E-05 |
| D0H4S114 | NM_001109988.1 | -7.00 | 2.07E-06 |
| Pcdh17 | NM_001013753.2 | -7.22 | 6.96E-04 |
| Sprr3 | NM_011478.2 | -7.24 | 1.44E-05 |
| Rn7sk | NR_030687.1 | -7.24 | 5.74E-04 |
| Klk8 | NM_008940.2 | -7.49 | 3.00E-05 |
| Fbln2 | NM_001081437.1 | -8.57 | 9.23E-07 |
| Cryab | NM_009964.2 | -8.97 | 7.91E-07 |
| Pip5kl1 | NM_198191.2 | -9.15 | 1.64E-04 |
| Acta1 | NM_009606.2 | -10.95 | 1.81E-04 |
| Tm6sf1 | NM_145375.3 | -18.36 | 4.43E-04 |
| Prrg3 | NM_001081135.1 | -26.55 | 9.13E-07 |
| Cdk14 | NM_011074.2 | -32.12 | 5.93E-05 |

**Supplemental Table 3. The Effects of ROCK1 and ROCK2 shRNA on the Expression of the Top VEGF Responsive Genes in MS1 Endothelial Cells**

| Gene Symbol | Accession Number | Fold Change [V *vs* C] | Fold Change [VR1 *vs* V] | Fold Change [VR2 *vs* V] |
|---|---|---|---|---|
| Cxcr4 | NM_009911.3 | 36.62 | -3.64 | -4.39 |
| Msmp | NM_001099314.1 | 9.50 | -4.44 | N/S |
| Hoxb13 | NM_008267.3 | 8.27 | -9.03 | -9.90 |
| 1600029D21Rik | NM_029639.2 | 8.00 | N/S | N/S |
| Kcne3 | NM_020574.5 | 7.39 | -4.44 | N/S |
| Sncaip | NM_026408.4 | 5.75 | -2.93 | -4.37 |
| 1810011O10Rik | NM_026931.2 | 5.47 | 2.07 | N/S |
| Lat2 | NM_020044.2 | 5.46 | N/S | -2.04 |
| Slc14a1 | NM_001171010.1 | 5.32 | -2.00 | -2.66 |
| Ngef | NM_001111314.1 | 5.26 | -11.50 | -12.63 |
| Rnf144a | NM_001081977.1 | 5.12 | N/S | -3.62 |
| St8sia4 | NM_001159745.1 | 5.09 | N/S | N/S |
| Pde2a | NM_001008548.4 | 5.06 | -2.20 | -3.57 |
| Kit | NM_001122733.1 | 4.93 | -2.91 | -2.04 |
| Nos2 | NM_010927.3 | 4.86 | N/S | -8.21 |
| Slc44a5 | NM_001081263.1 | 4.82 | -2.31 | -2.10 |
| Pgf | NM_008827.2 | 4.64 | N/S | -2.90 |
| Mir27a | NR_029746.1 | 4.56 | N/S | N/S |
| D930048N14Rik | NR_027958.1 | 4.54 | -2.00 | -2.30 |
| Chrnb1 | NM_009601.4 | 4.51 | -2.50 | -2.36 |
| Prelp | NM_054077.4 | 4.21 | -3.50 | -3.26 |
| Trib3 | NM_175093.2 | 4.15 | -2.10 | N/S |
| Csf1 | NM_001113529.1 | -4.65 | N/S | 2.30 |
| Fbln2 | NM_001081437.1 | -4.67 | N/S | -2.83 |
| Sema3c | NM_013657.5 | -5.02 | N/S | 3.34 |
| Ndst3 | NM_031186.2 | -5.14 | N/S | N/S |
| Npr3 | NM_001039181.1 | -5.15 | 2.80 | 3.26 |
| Timp3 | NM_011595.2 | -5.28 | 2.25 | 3.38 |
| Sepw1 | NM_009156.2 | -5.35 | 5.21 | 5.14 |
| Pspn | NM_008954.2 | -5.61 | N/S | 4.53 |
| Cyp1b1 | NM_009994.1 | -5.70 | N/S | 2.03 |
| 1700009N14Rik | NM_001081095.1 | -5.87 | N/S | 3.30 |
| Defb1 | NM_007843.3 | -6.50 | N/S | N/S |
| Ptplb | NM_023587.2 | -6.84 | N/S | N/S |
| Krt19 | NM_008471.2 | -6.87 | 6.13 | 16.19 |
| Mgat4c | NM_001162368.1 | -7.35 | 2.65 | N/S |
| Fhl1 | NM_001077361.1 | -7.95 | 5.07 | N/S |
| Olfr317 | NM_001011769.2 | -9.82 | N/S | N/S |
| Id2 | NM_010496.3 | -9.86 | 2.00 | 3.73 |
| Sprr3 | NM_011478.2 | -11.08 | 2.84 | N/S |
| Atoh8 | NM_153778.3 | -11.17 | 2.65 | 3.62 |
| Pip5kl1 | NM_198191.2 | -14.99 | N/S | N/S |
| Slc16a1 | NM_009196.3 | -15.45 | N/S | N/S |
| Acta1 | NM_009606.2 | -16.66 | N/S | N/S |
| Acta2 | NM_007392.2 | -21.96 | N/S | N/S |
| Id3 | NM_008321.2 | -42.71 | 6.23 | 14.21 |

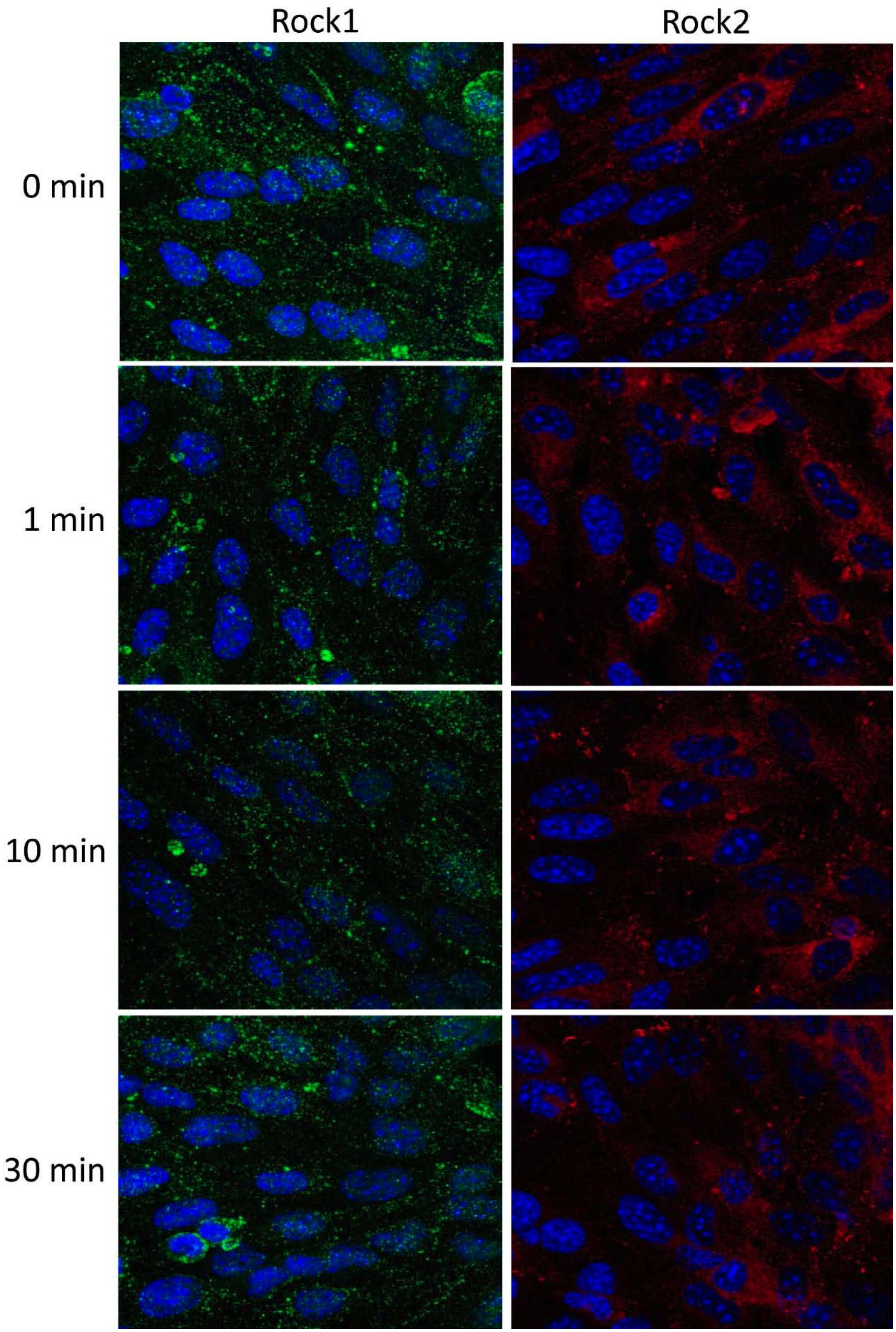


**Supplementary Fig. (1). ROCK1 & 2 localization is not affected by VEGF stimulation of endothelial cells.** Immunofluorescent detection of ROCK1 & 2 subcellular localization in MS1 endothelial cells at 0, 1, 10, and 30 minutes post-stimulation with 2.5 ng/ml VEGF using scanning confocal microscopy.
